# Filler-Dominated Polymer Nanocomposites: Molecular Transport in Extended Nanocavities

**DOI:** 10.1021/acs.jpcc.6c01646

**Published:** 2026-07-06

**Authors:** E. D’Amato, M Scarpa, R.S Brusa, P Battocchio, A Wagner, M.O Liedke, E Hirschmann, P Mengucci, A Korn, R Checchetto

**Affiliations:** † Department of Physics, University of Trento, Via Sommarive 14, Povo Trento 38123, Italy; ‡ Helmholz-Zentrum Dresden-Rossendorf, Institute of Radiation Physics,Dresden,Germany, Bautzner Landstr. 400, Dresden 01328, Germany; § TIPFA-INFN Trento, Vai Sommarive 14, Povo Trento 38123, Italy; ∥ Dept. SIMAU, Faculty of Engineering, Universit̀a Politecnica Delle Marche (UNIVPM), Via Brecce Bianche 12, Ancona 60132, Italy; ⊥ Department of Physics, Chemnitz University of Technology, Reichenhainer Str. 70, Chemnitz 09126, Germany

## Abstract

A deeper understanding of the molecular transport mechanisms in filler-dominated nanocomposites is a necessary step toward the development of innovative molecular separation membranes. Nanocomposite membranes were prepared with gas-impermeable Cellulose Nanocrystals (CNCs) at ∼80 wt % content and polyethylene glycol (PEG). Molecular transport takes place according to a configurational diffusion mechanism through extended nanocavities having a cross-sectional size of ∼0.52 nm between packed CNCs. The CO_2_ solubility-selectivity is favored by physical interaction with the ethylene oxide groups of dispersed PEGs. The PEG chain length affects the CO_2_ membrane permeability and selectivity, the first one maximized by low-molecular weight PEGs, the latter for polymeric PEGs.

## Graphical abstract

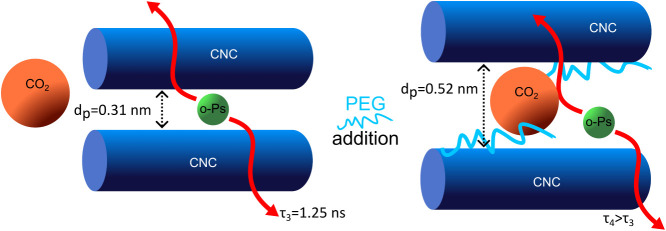

## 1 Introduction

The filler-dominated polymer nanocomposite is an innovative membrane architecture, promising enhanced molecular separation performances due to the presence of nanochannels acting as selective pathways for molecular diffusion.^1^ At present, several reports are available on nanocomposite membranes containing nanoporous fillers such as metal-organic frameworks (MOFs) or zeolites, and gas-impermeable two-dimensional (2D) fillers such as graphene, graphene oxide (GO), and MXenes.^1^ Different mechanisms control the mass transport process in the nanochannel diffusion pathways due to the complex interplay between polymer, filler, and penetrant molecules.^1,2^

In nanocomposite membranes with nanoporous fillers as dominating components, interconnected fillers form rigid nanochannels whose structure and tortuosity are defined by the filler porosity and distribution. Li et al. prepared nanocomposite membranes made of poly­(ethylene oxide) (PEO) containing 55 wt % zeolitic imidazolate framework-8 (ZIF-8), showing CO_2_selective transport due to size-sieving effects in the quasi-continuous porous filler network.^3^ Hua et al. suggested that size-sieving effects take place in 6FDA-DAM membranes with ZIF-8 content larger than 50 wt %, and the selective C_3_H_6_ transport was attributed to polymer–filler interaction affecting the ZIF-8 aperture size.^4^ Tan et al. prepared nanocomposite Matrimid membranes with 55 wt % content of Na-SSZ-39 porous nanoplatelets (sodium-exchanged aluminosilicate zeolite), which showed preferential CO_2_ transport due to the CO_2_-philicity of the nanofiller and molecular-sieving effects in the porous nanochannels.^5^

When gas- impermeable nanofillers are the dominant component of the membrane, ultrathin polymer layers form between the stacked nanofillers and act as diffusive nanochannels. The nanochannel structure, as well as the penetrant interaction with the local environment, controls molecular transport.^1,6–8^ In polymer–clay nanocomposite membranes made of styrene–butadiene rubber (SBR) and layered silicate clay, Wang et al. observed that clay largely reduced the penetrant permeability by forming tortuous diffusional paths and decreasing the free volume of the polymer layers.^6^ Liu et al. prepared POSS/PDMS nanocomposites (POSS: C_8_H_24_O_12_Si_8_ nanoparticles with sizes of 200–300 nm) and attributed the large butanol/water selectivity to attractive filler–polymer interactions, which opened the voids in polymer nanochannels that favor the transport of large-sized molecules.^7^ Shen et al. prepared neat MXene nanofilms with preferential H_2_ transport but observed that borate and polyethylenimine (PEI) molecules interlocked into MXenes selectively enhanced the CO_2_ transport rates by changing the transport mechanism from “diffusion-controlled” to “solution-controlled”.^9^ Ding et al. reported, on the contrary, that the CO_2_ permeability of MXene nanolaminates was lower than that of N_2_ and comparable to the CH_4_ one, as a consequence of the strong interaction of migrating CO_2_ molecules with the oxygen-containing groups on the MXene surface.^10^

Cellulose Nanocrystals (CNCs) form an alternative class of nonporous organic nanomaterials used in nanocomposite membranes for molecular separation.^11^ CNCs are rod-shaped nanostructures with a diameter of a few nanometers and a length in the 100 to 1000 nm range, obtained from cellulose fibrils by removing the disordered regions. CNCs expose hydrophobic and hydrophilic crystallographic planes, where the former are responsible for the tight packing of the nanocellulose by van der Waals interactions, while the hydrophilic planes expose about 5.4 −OH groups per nm^2^ and are excellent hydrogen bond donors and acceptors.^12^ Due to their peculiar physicochemical features, CNCs have been deeply studied as nanostructures for the preparation of advanced molecular separation membranes.^13^ The neat CNC membranes’ separation mechanism is correlated to the CNC network arrangement and porous structure, thus making them attractive for different separation applications.^13–15^ We observed that a few micrometer-thick CNC films act as an impermeable barrier for CO_2_, N_2_, and O_2_ but permit the selective transport of the small-sized *D*_2_ and *He* molecules.^16,17^ Structural analysis revealed that CNCs undergo a self-organization process, forming elongated, rigid nanocavities between the aligned CNCs. Molecular diffusion occurs in the nanochannels formed by these interconnected nanocavities, and the *D*_2_ and *He* selective transport results from size-sieving effects.^16^ The dispersion of PEG400 oligomers between the CNC units enhances the CO_2_, N_2_, and O_2_ transport properties. The membrane permeability increases with the PEG content, and the CNC/PEG nanocomposite membranes resulted CO_2_ selective^18^ due to the Lewis acid–base interactions between the CO_2_ penetrant and the PEG ether groups.^19,20^

However, despite the promising properties of CNC as a nanofiller and the envisaged application of the obtained composites,^21,22^ their use as a dominating filler component of nanocomposite membranes for gas separation has not been investigated up to now.

Herein, we present an experimental study on molecular transport through filler-dominated CNC/PEG nanocomposite membranes. The PEG size varied within the macro-organic chemistry range, from a low molecular weight oligomer (PEG400) to small-sized polymers (PEG 8000 and PEG 20000). It is noteworthy that, in this size range, significant changes in physical and structural properties are expected, as the rigid oligomers and the flexible small polymers undergo different inter- and intramolecular interactions. The variable physical characteristics of the organic matrix could change the membrane architecture while maintaining the same chemical interactions between the two membrane components and the permeant molecules. CNCs and PEGs are expected to contribute to the gas transport properties in different ways, as CNCs form a network of nanochannels, and the soft PEG molecules, besides changing the membrane architecture, promote or inhibit the motion of the permeant molecules by surface interactions within the porous framework. The investigation of these effects is highly advised for the development of innovative gas separation membranes.^23^

## 2 Methods

### 2.1 Nanocomposite Membrane Preparation

CNC/PEG polymer nanocomposites used in this study were prepared according to the following procedure. Cellulose nanocrystals (CNCs) with carboxylate charged group content of 1.8 μmol/mg were obtained by TEMPO-mediated oxidation of cellulose pulp provided by SCA (Sundsvall, Sweden) using the procedure reported by Bettotti et al.^24^ The *Z*-potential of CNC suspensions diluted in deionized water was measured by a Litesizer apparatus (Anton Paar) and was −46 ± 4 mV. AFM images of the CNC suspension deposited on a silicon slab showed the typical rod shape with a length in the 80–250 nm range and a width of 3.0 ± 0.5 nm.^16^ The PEG/CNC composites were made by mixing CNC suspensions (8 mg mL^–1^) and aqueous solutions of PEG with molecular weights of 400, 8000, and 20000 Da (Sigma-Aldrich) to obtain a weight ratio 21 ± 1 wt % PEG/CNC. Then, the CNC or CNC/PEG suspensions were stirred for 2 h at room temperature under mild vacuum and cast on polystyrene Petri dishes. Films were obtained by drying the deposited suspension at 37 °C for 48 h. They were then peeled from the Petri support and stored over molecular sieves. The mass density per unit area of the final films was 60 ± 10 μg/mm^2^. The 21 ± 1 wt % PEG content was chosen because it ensures high CO_2_ selectivity and resonable transport rates within a filler- dominated structure. In a previous study carried out using PEG400 at a lower content (i.e., 14 wt %), the CNC/PEG membrane was CO_2_ selective but the CO_2_ transport rates, 0.42 ± 0.04 Barrer, were too low for practical applications.^18^

### 2.2 Characterization

Scanning electron microscopy (SEM) observations of the nanocomposite membrane samples were carried out with a Zeiss Supra 40 Field Emission SEM (FE-SEM) equipped with a Bruker Z200 microanalysis system. Images were acquired by the in-lens high-resolution detector using samples coated with a thin Cr layer. Cross-sectional analyses were carried out on samples that were fractured after immersion in liquid nitrogen.

Differential Scanning Calorimetry (DSC) tests were carried out in the −60 to 80 °C using a PerkinElmer DSC8500 apparatus. A first heating run at 5 °C/min was performed to eliminate the sample’s thermal history, followed by a cooling run at 10 °C/min, and finally, a second heating run at 5 °C/min was recorded.

The void structure of the CNC/PEG nanocomposites was studied by depth-resolved Positron Annihilation Lifetime Spectroscopy (PALS) with monoenergetic positron (*e* ^+^) beams at the Elbe positron source (EPOS),^25,26^ see the Supporting Information section for details. We carried out experiments with *e* ^+^ implantation energy ranging from 1 to 11 keV, resulting in a mean implantation depth ẑ from 150 to 1200 nm.^27^ Positrons injected into polymers thermalize, and a fraction of them forms positronium (*Ps*)—the positron-electron bound state—which exists in two configurations: parapositronium (*pPs*) (singlet state with a mean vacuum lifetime of 125 ps) and orthopositronium (*oPs*) (triplet state with a maximum vacuum lifetime of 142 ns). *oPs* can be trapped in nanometer-sized regions of lower electron density, such as voids, where the positron of *oPs* annihilates by exchanging the positron with a surrounding electron having opposite spin (“pick-off” process). The *oPs* lifetime is then reduced to several nanoseconds, and its value can be related, through quantum models, to the size and geometry of the voids where the pick-off process occurs.^28^ The lifetime values (*τ*_*i*_) of the free *e* ^+^ and *oPs,* along with their intensities (*I*_*i*_) were obtained from the measured positron annihilation spectra *F*(*t*) analyzed by the PATFIT package.^29^ We focused our analysis on the long-lifetime components (*τ*_3_ and *τ*_4_) that provide information on the average size of the voids where penetrant diffusion occurs, while the related intensity is proportional to the density of these voids.^30^ Conversely, we did not analyze the trend of *τ*_1_ and *τ*_2_ which are associated with the annihilation of parapositronium and free positrons in the bulk.

### 2.3 Gas Transport Tests

Gas transport tests of the CNC/PEG nanocomposite membranes were carried out using the gas phase permeation technique under single gas conditions and a dead-end configuration, employing disc-shaped samples with a 13 mm diameter and thickness *L* ranging from 35 to 55 μm, as measured by a micrometer. We used as test gas *α* nitrogen (N_2_), carbon dioxide (CO_2_), and methane (CH_4_), as test gases (see Table 1) and performed permeation tests at temperatures between 25 and 77 °C. Briefly, the membrane sample separates the feed chamber from the permeate chamber. Before each permeation test, the feed chamber was evacuated to a final pressure of ∼10^–2^ mbar. At time *t* 0, the feed side of the membrane is exposed to the test gas *α* at feed pressures ∼10^3^ mbar. Gas molecules permeated through the membrane to the continuously pumped permeate chamber (where the background pressure was in the low 10^–8^ mbar range). The permeation flux *f*_*α,exp*_(*t*) of the *α* test gas was monitored under transient and stationary transport conditions by measuring its partial pressure *p*_*α*_(*t*) in the permeate chamber by a calibrated Quadrupole Mass Spectrometer (QMS).^31^ The obtained *f*_α,exp_(*t*) curves are analyzed in the framework of the solution-diffusion model to evaluate the gas permeability *P*_*α*_ and the effective gas diffusivity *D*_*α*_.^32^ The gas solubility *S*_*α*_ is then evaluated by the relation *S*_*α*_ = *P*_*α*_/*D*_*α*_.^32^ Information on the experimental apparatus, measurement procedure, and analysis of the *f*_α,exp_(*t*) curves can be found in the Supporting Information section (Figure S1, Supporting Information) and in a previous paper.^31^

**Table 1:** Physical and Chemical Properties of Gases Involved in Carbon Dioxide Separation.^32,38^ ^a^

Gas	molecular weight (g/mol)	critical temperature (K)	Lennard-Jones diameter (nm)	kinetic diameter (nm)	*ε*/*k*_*B*_(K)
CO_2_	44.01	304	0.394	0.33	195
N_2_	28.01	126	0.379	0.364	71
CH_4_	16.04	190.5	0.376	0.38	149

a The *ε* parameter is the energy constant in the Lennard-Jones potential, while *k*_B_ is the Boltzmann constant.

## 3 Results and Discussion

Figure 1 reports the surface (upper row) and the cross-sectional (lower row) FE-SEM images of the nanocomposite membranes with 21 wt % PEG400, PEG8000, and PEG20000, from left to right, respectively. It can be observed that the prepared CNC/PEG nanocomposites exhibit uniform surface morphology without macroscopic defects and that their cross-sections show an arrangement in a layered fashion. CNC and PEG are not distinguishable, and no PEG aggregates are detectable, suggesting that PEG is well-dispersed between the CNC units. Compared to the neat CNC membranes (Figures S2 and S3, Supporting Information), the present nanocomposites exhibit a less regular alignment of the CNCs,^16^ showing that the interaction between CNCs and the dispersed PEG chains reduces the order in the membrane nanoscale morphology.^33,34^ The similar structure of the neat CNC and composite CNC/PEGs suggests that, in the present nanocomposite samples, the interaction with PEG does not significantly shield the repulsion between the highly carboxylated CNCs. The packing of CNC units in a laminated fashion and the absence of the chiral nematic structures in the present nanocomposites, which was observed also in materials prepared with different procedures,^35,36^ could be ascribed to the repulsive forces between the highly charged CNCs at the low salt concentration of the CNC-PEG suspensions used in the present work.^37^

**Figure 1:**
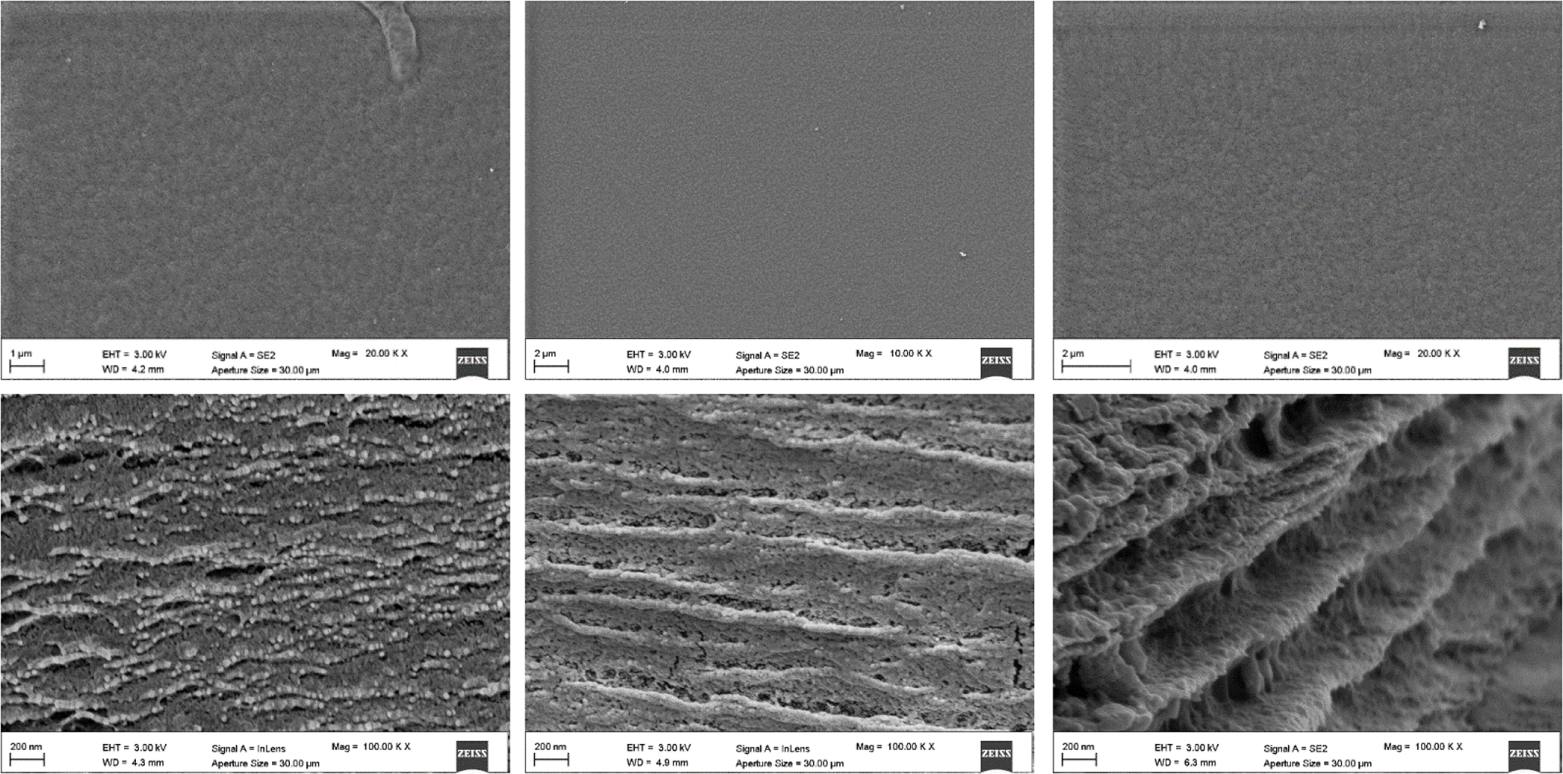
FE-SEM images of the surface (upper row) and cross-sectional (lower row) views of the CNC/PEG membranes with PEG400, PEG8000, and PEG20000, from left to right, respectively.

The thermograms of the first cooling run, line (a), and the second heating run, line (b), are reported in Figure 2.

**Figure 2:**
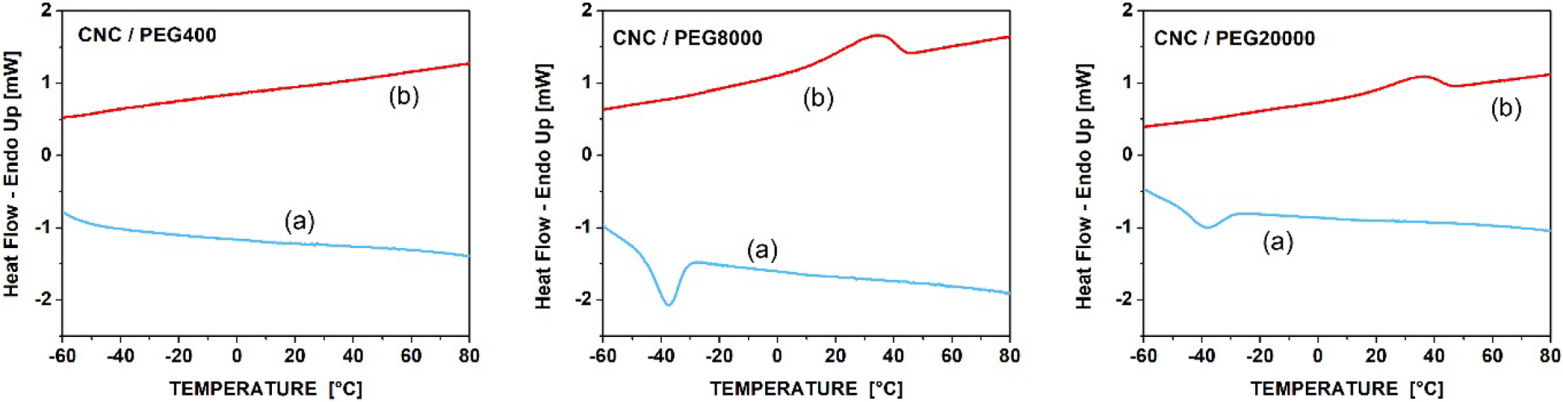
DSC thermograms of the nanocomposite PEG/CNC membranes with PEG400, PEG8000, and PEG20000, from left to right, respectively. Lines (a): first cooling run. Lines (b): second heating run. In the DSC tests, we used 5.48 mg of CNC/PEG400, 7.25 mg of CNC/PEG8000, and 4.42 mg of CNC/PEG20000.

The cooling runs of the CNC/PEG8000 and CNC/PEG20000 nanocomposite membranes show a peak at −40 °C. Their heating runs show an inflection point at −40 °C and an endothermic peak at 35 °C. This peak is attributable to the melting of PEG domains but occurs at temperatures lower than those of bulk PEG8000 (i.e., 50 °C) and bulk PEG20000 (i.e., 63 °C). Similarly to the neat CNC nanocomposite membrane, no DSC signal is observed in the heating and cooling runs of the CNC/PEG400 nanocomposite membrane (Figure S4, Supporting Information). This finding indicates that a fraction of PEG8000 and PEG20000 macromolecular chains dispersed in the nanocomposite form nanosized domains with low crystalline degree, which are not detected by SEM imaging. Note that the CNC/PEG composites with carboxylated CNC behave similarly to those containing CNCs produced by sulfuric acid hydrolysis.

In Figure 3, we report the gas permeability *P*_*α*_ = *D_α_S*_*α*_ (values are reported in Barrer, 1 Barrer = 10^–10^ cm^3^(STP)/cm^3^ cmHg = 7.6 × 10^–14^ cm^3^(STP)/cm^3^ Pa) and diffusivity *D*_*α*_ while in Figure 4 we report the gas solubility *S*_*α*_.

**Figure 3:**
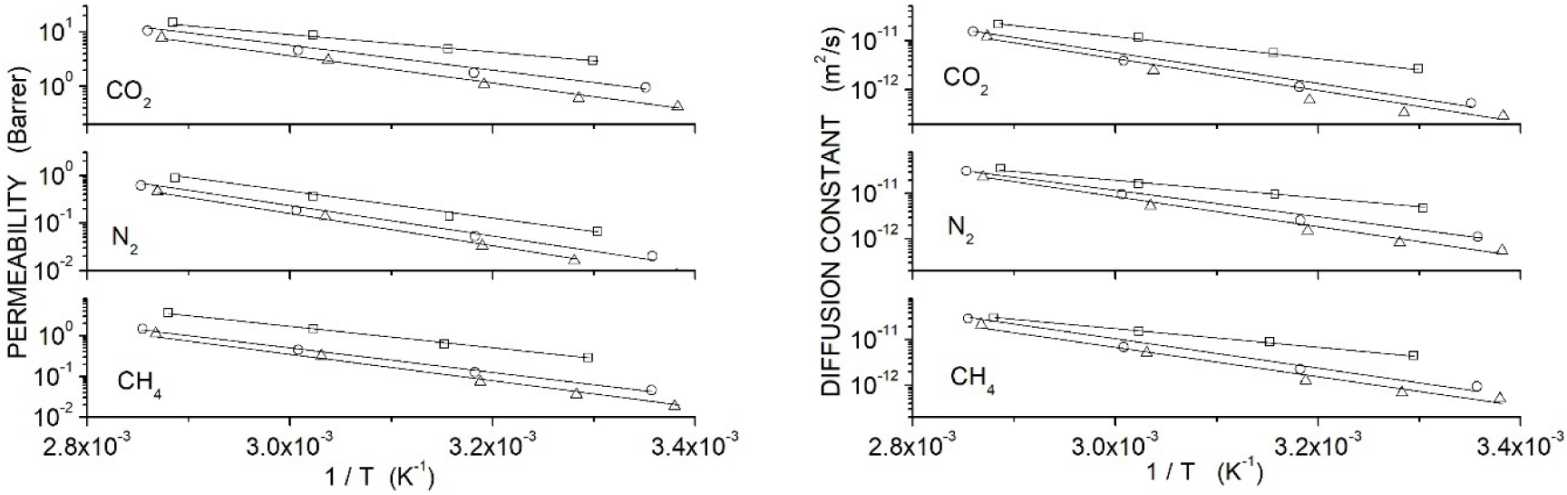
Arrhenius plot of the permeability *P*_*α*_ and diffusivity *D*_*α*_ values of the nanocomposite membrane for the examined test gases. Squares are pertinent to the CNC/PEG400 membrane, triangles to the CNC/PEG8000 membrane and circles to the CNC/PEG20000 membrane. Experimental indeterminations are inside the size of the symbols.

**Figure 4:**
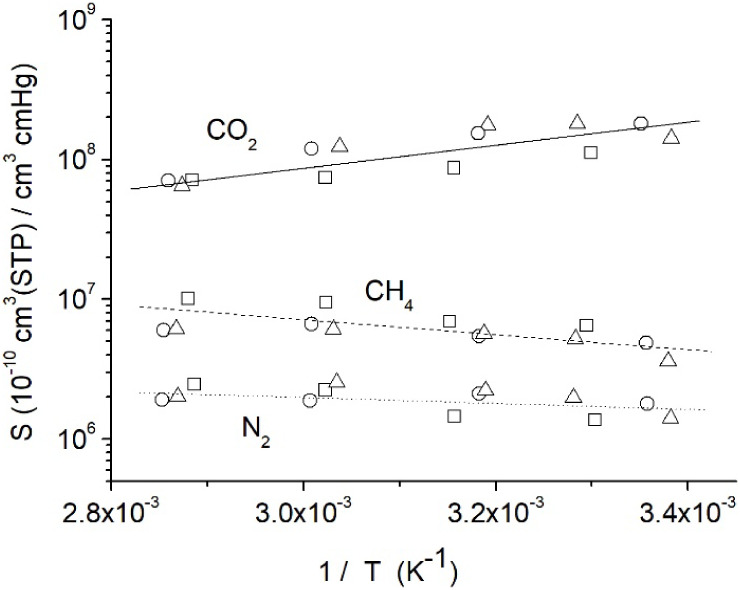
Arrhenius plot of the gas solubility values: (□) CNC/PEG400 membrane, (Δ) CNC/PEG8000 membrane, and (Ο) CNC/PEG20000 membrane. Experimental uncertainties are within the size of the symbols. Lines are provided as guides for the eye: solid line for CO_2_ data, dashed for CH_4_ data and dotted line for N_2_ data [10^–10^ cm^3^(STP)/cm^3^ cmHg = 7.6 × 10^–14^ cm^3^(STP)/cm^3^ Pa = 3.39 × 10^–18^ mol/cm^3^ Pa].

Figure 3 shows that, with increasing temperature, *P*_*α*_ and *D*_*α*_ increase with thermally activated behavior. Conversely, the solubility values *S*_*α*_ of the CH_4_ and N_2_ penetrants are, inside their experimental indetermination, nearly constant while the solubility values of the CO_2_ penetrant slowly decrease increasing temperature. The effective values of activation energy for permeation, *E*_*P*_, and for diffusion, *E_D_* are found by fitting the transport data to the Arrhenius equation and are reported in Table 2. Looking at this table, we note that in each nanocomposite membrane: (i) the *E*_*P*_ value for CO_2_ is lower than the *E*_*P*_ value for N_2_ and CH_4_ which are almost equal; (ii) the *E*_*D*_ values are equal for the three gases, within the limits of experimental accuracy.

**Table 2:** Effective Values of the Activation Energy for Permeation *E*_*P*_and Diffusion *E*_*D*_ in the CNC/PEG Membrane Samples

	CNC/PEG400		CNC/PEG8000		CNC/PEG20000	
Penetrant molecule	*E*_*p*_ [kJ/mol]	*E*_*D*_ [kJ/mol]	*E*_*p*_ [kJ/mol]	*E*_*D*_ [kJ/mol]	*E*_*p*_ [kJ/mol]	*E*_*D*_ [kJ/mol]
*CH*_4_	52 ± 2	38 ± 1	68 ± 2	63 ± 5	58 ± 3	56 ± 6
*N*_2_	52 ± 2	39 ± 2	67 ± 2	62 ± 4	56 ± 3	56 ± 4
*CO*_2_	33 ± 1	42 ± 1	49 ± 2	63 ± 7	41 ± 3	56 ± 6

Considering the PEG size, from Table 2, we observe that (i) *E*_*D*_ values are equal, within their experimental uncertainty, in the nanocomposite membranes containing polymeric PEGs, and (ii) *E*_*D*_ values of CNC/PEG8000 and CNC/PEG20000 nanocomposites are larger than *E*_*D*_ values of the CNC/PEG400 nanocomposite. PEG400 is a liquid with high affinity for the CNCs in the investigated temperature range, and DSC analysis shows that it is better dispersed than polymeric PEGs, which, conversely, form nanoaggregates. This shows that the gas transport properties of the CNC/PEG nanocomposite membranes exhibit a discontinuity when transitioning from the class of oligomers to the class of polymers, suggesting that the transport properties of the CNC/PEG nanocomposite membranes depend on the membrane architecture, as it results from the CNC structuring and PEG assembly, not just on the PEG molecular weight and size.

Neat CNC membranes selectively transport *D*_2_ and *He* molecules by size-selective mechanism through diffusive paths formed by the elongated nanocavities between the packed CNCs^16^: the increased CO_2_, CH_4_, and N_2_ transport rates of the nanocomposite PEG/CNC membranes suggest that PEG dispersed in the nanocavities form voids permitting the transport of the CO_2_, CH_4_, and N_2_ molecules

Figure 5 shows the long-lifetime and relative intensity values obtained with (i) neat CNC and CNC/PEG400 membranes at 23 °C and different implantation depths (first row), (ii) CNC/PEG400 membranes at 11 keV *e* ^+^ implantation energy increasing the sample temperature (central row) and (iii) neat CNC and CNC/PEG nanocomposite membranes at 11 keV *e* ^+^ implantation energy and 23 °C while changing the PEG molecular weight (last row). The set of PALS data is reported in Table S1–S5 of the Supporting Information. The long lifetimes (*τ*_3_ and *τ*_4_) and the corresponding intensities (*I*_3_ and *I*_4_) values of CNC/PEG8000 and CNC/PEG20000 nanocomposite membranes exhibit the same behavior as the long lifetimes and intensities of the CNC/PEG400 membrane when varying the positron implantation depth and nanocomposite temperature.

**Figure 5:**
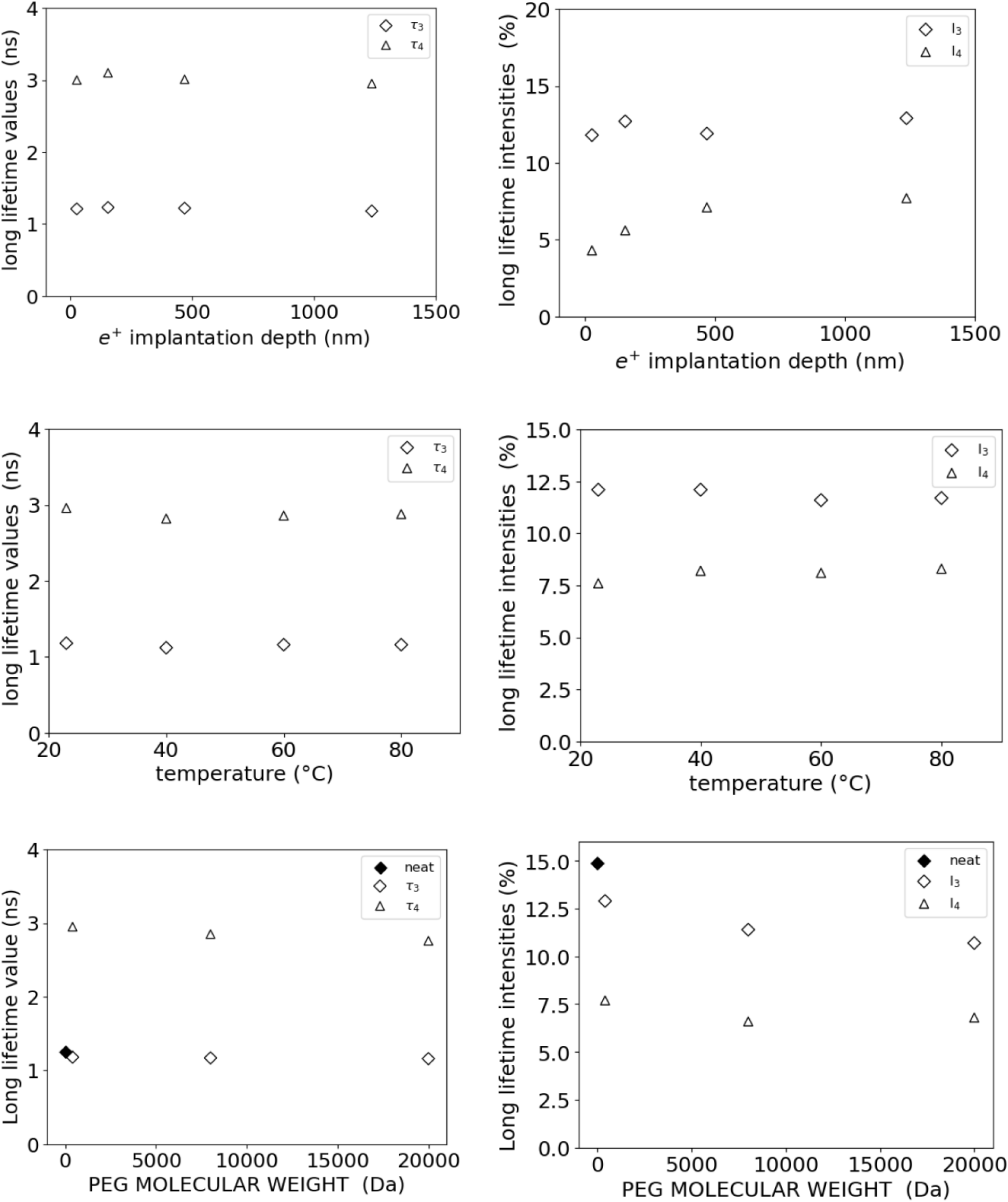
Long- lifetime components *τ*_3_ (open diamonds) and *τ*_4_ (open triangles) and relative intensity values *I*_3_ (open diamonds) and *I*_4_ (open triangles) of the CNC/PEG nanocomposite membranes. Upper row: CNC/PEG400 membrane, 23 °C, at different *e* ^+^ implantation depths. Central row: CNC/PEG400 membrane, 11 keV *e* ^+^ implantation energy, from 23 to 80 °C. Lower row: 11 keV *e* ^+^ implantation energy, 23 °C, neat CNC (solid symbols) and CNC/PEG membranes (open symbols). The implantation depth was evaluated using a CNC density value of 1.5 g/cm^3^. Experimental uncertainties are within the size of the symbols.

The neat CNC membranes show a single long-lifetime component *τ*_3_ = 1.25 ± 0.01 ns with relative intensity *I*_3_ ∼ 14%, independently of the positron implantation energy. It was attributed to the *oPs* annihilation in void regions between the packed cellulose nanocrystals, which we modeled as elongated nanocavities with a cross-sectional size *d*_*p*_ ∼ 0.31 nm.^16^ This size agrees with the gas barrier properties of the neat CNC membranes, which are selectively permeable to the small H_2_ and *He* molecules.^16,17^ The *τ*_3_ value of 1.25 ± 0.02 ns observed in the present neat CNC membranes is lower than that of the chiral nematic films, in accord with the structures observed by SEM, i.e., aligned CNCs in the present samples and helices in the nematic films.^39^

$$\tau_{4}^{-1}=\lambda_{0}\left[1-{\left(\frac{d_{p}}{d_{p}+2\Delta R}+\frac{1}{\pi }sin\frac{\pi d_{p}}{d_{p}+2\Delta R}\right)}^{2}\left(\frac{L_{cavity}}{L_{cavity}+2\Delta R}+\frac{1}{\pi }\sin\frac{\pi L_{cavity}}{L_{cavity}+2\Delta R}\right)\right]$$

The best fit of PALS spectra of nanocomposite CNC/PEG membranes provided: (i) slightly reduced *τ*_3_ and *I*_3_ values with respect to those of the neat CNC and (ii) a fourth long lifetime component *τ*_4_ > *τ*_3_ with intensity *I*_4_ < *I*_3_, see Figure 5. Figure 5 also shows that the *τ*_3_ and *τ*_4_ lifetime values and corresponding *I*_3_ and *I*_4_ intensities have negligible variation changing the *e* ^+^ implantation depth, the PEG molecular weight and the nanocomposite temperature.

The nanocomposite PEG/CNC membranes show same CNC arrangement with PEG chains dispersed between the CNC structural units: the *τ*_4_ component, which is accompanied by the *τ*_3_ one, points out that in the CNC/PEG nanocomposites a fraction of the elongated nanocavities have cross-sectional size larger than the 0.31 nm observed in the neat CNC membrane. To evaluate their cross-sectional size, we model these “enlarged” elongated nanocavities as prisms with square cross section *d*_*p*_ and length *L*_*cavity*_ = *md*_*p*_(*m* ≫ 1).^16^ For this prism-like geometry, the *d*_*p*_ value can be obtained from the measured *τ*_4_ data by the relation:^28^ where *λ*_0_ ≃ 0.5 ns^–1^ is the *oPs* annihilation rate in the bulk state and Δ*R* = 0.166 nm is an empirical parameter describing the electron layer thickness.^28^ This equation takes input values the nanocavity length *L*_*cavity*_ and cross-section *d*_*p*_ returning the expected *oPs* lifetime value. For elongated nanocavities (*m* ≫1), the experimentally obtained *τ*_4_ value can be reproduced with *d*_*p*_ = 0.52 nm. To show this point, in Figure 6 we report the calculated *τ*_4_ value (see solid circles) as a function of the normalized nanocavity length $m=\frac{L_{cavity}}{d_{p}}$ : the two dotted lines mark the range of the measured *τ*_4_ values reported in Figure 5. Note that the obtained cross-section *d*_*p*_ = 0.52 nm is larger than the size of all permeating molecules, see Table 1

**Figure 6:**
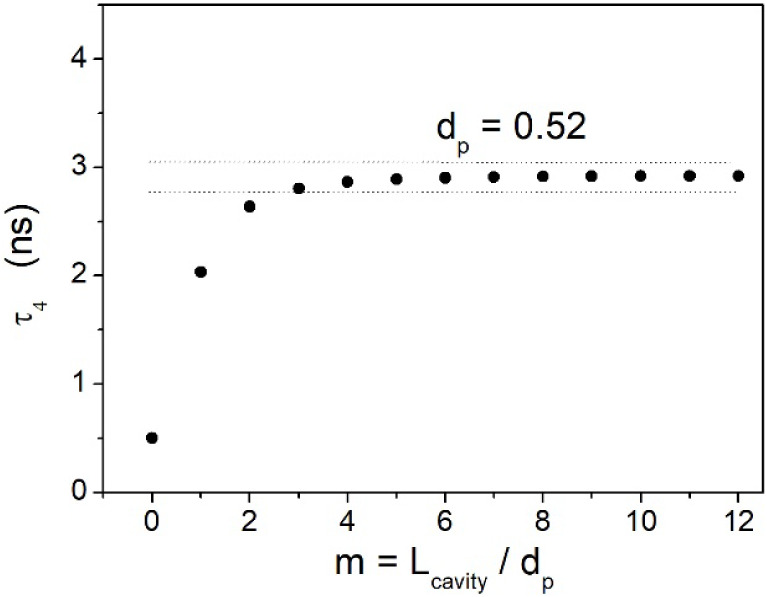
Calculated value of the long-lifetime component *τ*_4_ as a function of the normalized nanocavity length m *L_cavity_*/*d_p_* for *d*_*p*_ = 0.52 nm. Horizontal dotted lines describe the interval values of the experimentally measured *τ*_4_ parameter; see Figure 5.

PALS measurements show that the enlarged nanocavities are uniformly distributed along the membrane thickness, thus permitting the formation of diffusive pathways for permeating molecules. The enlarged nanocavities have an open volume structure similar to that of glassy polymers; in fact, neither their size (see *τ*_4_ values) nor their density (see *I*_4_ values) change with temperature reasonably because PEG confinement between CNCs units limits the molecular thermal fluctuations. Consequently, the thermally activated behavior of molecular diffusion cannot be attributed to the opening with temperature of the nanocomposite free volume, where penetrant diffusion occurs, as in polymer- based membranes.^40^ When the size of the elongated nanocavity *d*_*p*_ is comparable to the size of the diffusing molecule, as in the present CNC/PEG nanocomposite membranes—see Table 1—the thermally activated behavior of the diffusion constant indicates that transport occurs in a configurational regime.^41^ Diffusing molecules are hosted in specific sites on the nanochannel wall surface and, similarly to surface diffusion processes, jump between neighboring sites.^41^ The larger gas diffusivity and permeability of the CNC/PEG400 membrane is a consequence of the better dispersion of this low molecular weight PEG between the CNC units. DSC analyses indicated, in fact, that PEG400 does not form aggregates, thus promoting the formation of a uniformly distributed diffusive network. The consequence is a larger effective molecular diffusivity and a lower effective activation energy for diffusion than in the CNC/PEG8000 and CNC/PEG20000 nanocomposite membranes (see Table 2).

The ideal selectivity values $\alpha_{P}\left(CO_{2}/N_{2}\right)=\frac{P_{CO2}}{P_{N2}}=\frac{D_{CO2}}{D_{N2}}\frac{S_{CO2}}{S_{N2}}$ and $\alpha_{P}\left(CO_{2}/CH_{4}\right)=\frac{P_{CO2}}{P_{CH4}}=\frac{D_{CO2}}{D_{CH4}}\frac{S_{CO2}}{S_{CH4}}$ are reported in Figure 7 as a function of temperature. In these figures, the diffusivity-selectivity terms $\alpha_{D}\left(CO_{2}/N_{2}\right)=\frac{D_{CO2}}{D_{N2}}$ and $\alpha_{D}\left(CO_{2}/CH_{4}\right)=\frac{D_{CO2}}{D_{CH4}}$ and the solubility-selectivity term $\alpha_{S}\left(CO_{2}/N_{2}\right)=\frac{S_{CO2}}{S_{N2}}$ and $\alpha_{S}\left(CO_{2}/CH_{4}\right)=\frac{S_{CO2}}{S_{CH4}}$ are also reported.

**Figure 7:**
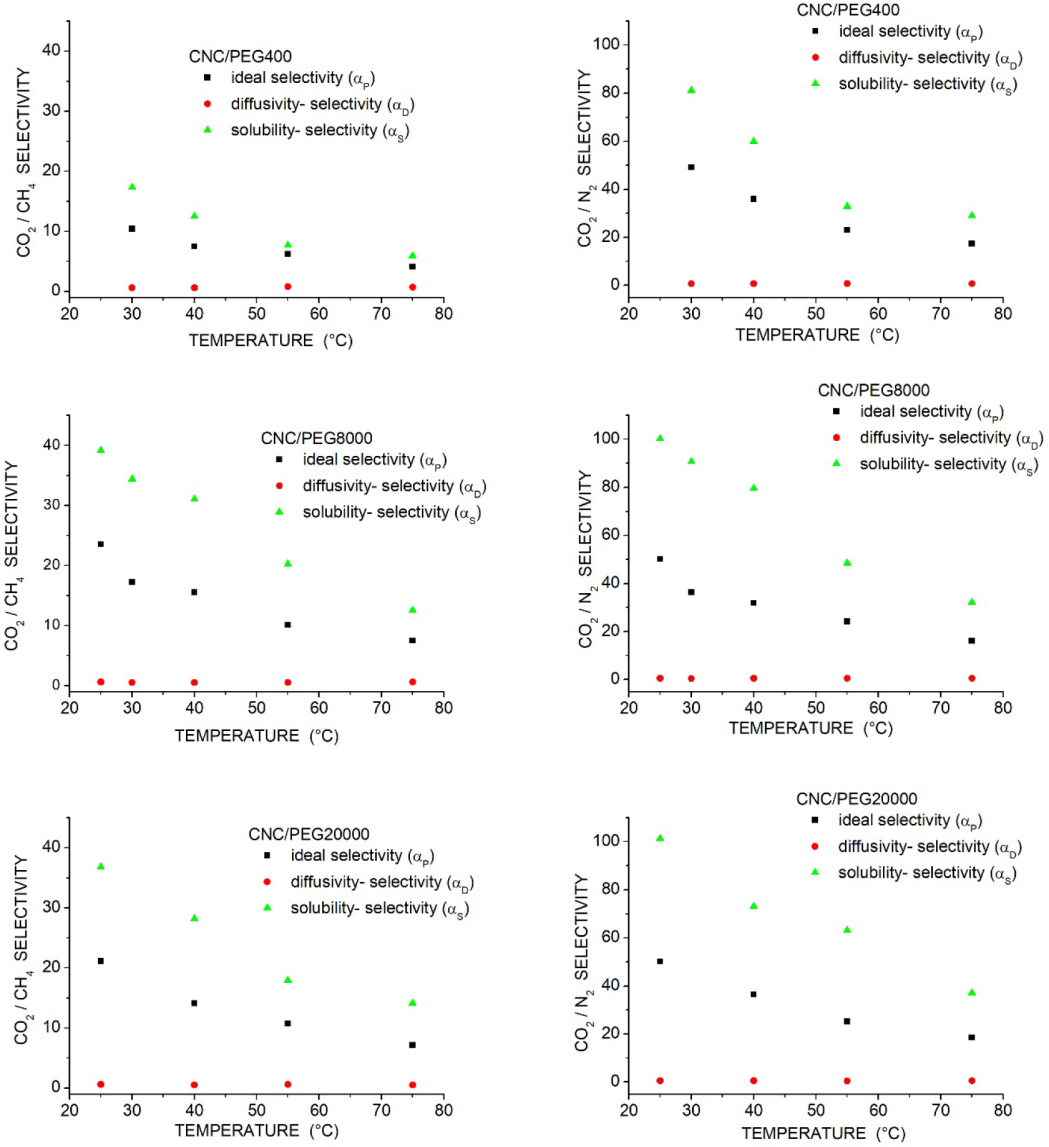
Ideal CO_2_/CH_4_ (left) and CO_2_/N_2_ (right) selectivity values. Upper panel: CNC/PEG400 membrane. Central panel: CNC/PEG8000 nanocomposite membrane. Lower panel: CNC/PEG20000 nanocomposite membrane.

From the data in Figure 7, we can observe that in all CNC/PEG membranes and at any temperature, the ideal CO_2_ selectivity has a solubility-selective character. The diffusivity selectivity is, in fact, close to 0.5 and does not change with temperature. The nondiffusive character of the membrane selectivity is compatible with the void structure of the enlarged extended nanocavities, as revealed by PALS analysis, which presents a cross-sectional size large enough to accommodate all permeating molecules and is independent of PEG molecular weight and temperature.

The CO_2_ solubility-selectivity in polymers is generally attributed to the higher CO_2_ condensability^32^ and in the PEG/CNC, it is favored by specific interactions between the quadrupolar moment of the CO_2_ molecule and the dipole moment of the PEG ether group,.^42,43^ PEG contains, in fact, oxygen atoms at every third position of the molecular backbone, with an average dipole moment per constitutional repeating unit of 1.040 ± 0.002D and two electron pairs on the oxygen atoms.^19^ PEG thus acts as an effective electron donor and a good hydrogen bond acceptor, with an angle of the C–O–C group of 110°, which restricts the dipole moment to preferentially interact with condensable gases.^20^

Figure 7 shows that the CO_2_ solubility-selectivities *α*_*S*_(CO_2_/CH_4_) and *α*_*S*_(CO_2_/N_2_) decrease increasing temperature: in the CNC/PEG8000 nanocomposites, for example, *α*_*S*_(CO_2_/CH_4_) decreases from ∼40 at 27 °C to ∼15 at 77 °C. This trend can be explained by observing that the CH_4_ and N_2_ solubilities *S*_N2_ and *S*_CH4_, in all CNC/PEG nanocomposite membranes, remain nearly constant with increasing temperature, while *S*_CO2_ shows a slight decrease (see Figure 4.

Comparing the different PEG formulations, we observe a competition between The the opposite permeability versus selectivity trend, which could be a consequence of the formation of PEG8000 and PEG200000 nanoaggregates: on one side, they hinder the formation of a uniform diffusion network for all the gas molecules; on the other side, they provide an interaction site with a large surface-to-volume ratio specific for the CO_2_ molecules.

Experimental data in Figure 3 show that in the present filler-dominated CNC/PEG nanocomposite membranes, the CH_4_ diffusivity is larger than the CO_2_ one. This was experimentally observed in other nanoporous systems^23^ as, for example, MOF-5 and Zeolite 5A,^44^ and H-ZSM-5,^45^ and was also calculated by Molecular Dynamics simulations in MFI zeolites^46,47^ and in ZIF-8 ^48^ and anthracite.^49^ The above-cited diffusivity values for CH_4_ are a factor ∼2 larger than the diffusivity values for CO_2_, as observed in the present study. Molecular transport through nanochannels is controlled by the complex interplay of mechanisms acting at the nanoscale level.^23,50^ The nanochannel geometry and penetrant size/shape control molecular confinement and molecular sieving effects.^23,50^ The physical interaction of diffusing molecules with the nanochannel walls controls penetrant adsorption.^23,50^ CO_2_ is a linear molecule with quadrupole moment while CH_4_ is a nonpolar, symmetric one. In our CNC/PEG nanocomposites, we thus expect that CO_2_ molecules experience stronger interactions with the PEG’s ethylene oxide groups than CH_4_, thus slowing down the CO_2_ diffusivity.

Many studies report on the separation properties of Mixed Matrix Membranes (MMMs) incorporating PEGs, where the polymer is the dominating component. These studies show that PEGs play a dual role: (i) the R–O–R’ polar ether groups of the flexible PEG chains improve the CO_2_ solubility due to their strong affinity for CO_2_ molecules, and (ii) PEG acts as a compatibilizer, mitigating filler–matrix interface defects and preventing filler aggregation.^42^ Conversely, only a few studies report on the gas separation properties of filler-dominated nanocomposite membranes incorporating PEGs,^3,51^ . Luo et al. reported that the CO_2_ preferential transport through filler-dominated gas-impermeable MXenes/PEG600 nanocomposite membranes takes place between the layered MXenes nanosheets with dispersed PEG chains via a solution-diffusion mechanism. They attributed the CO_2_ selectivity to CO_2_ interaction with the PEGs’ CO_2_-philic groups.^51^ Studies with porous fillers as dominating components were carried out by Chen et al. using Cu­(SiF_6_)­(pyz)_3_ MOF^2^ and by Li et al. using ZIF-8.^3^ They observed that the CO_2_ preferential transport was due to size-sieving effects in nanochannels formed by the interconnected porous fillers, with PEG acting as a binder. These studies have also shown that with increasing temperature, the gas transport rates improved, but the CO_2_ membrane selectivity decreacsed.^3,51^ These effects were attributed to the opening of the membrane free volume with temperature, which favors the transport of larger-sized CH_4_ and N_2_ molecules.^51^

Contrary to the above-cited works,^3,51^ in our CNC/PEG nanocomposites, the extended nanocavities between the packed CNC units do not change cross-sectional size with changing temperature. Nevertheless, we also observe that CO_2_ selectivity decreases with temperature. In our sample, this is because the CO_2_ solubility decreases while the solubility of other penetrant molecules remains constant as the temperature increases. Molecular transport takes place via a configurational diffusion mechanism, and the preferential CO_2_ transport results from CO_2_ interaction with PEG ether oxide groups, improving the CO_2_ solubility. The CO_2_ solubility with increasing temperature could be attributed to the weakening of the CO_2_ interaction with PEGs.

## 4 Conclusion

In neat CNC membranes, the selective H_2_ and *He* transport takes place through nanochannels formed by interconnected elongated nanocavities between packed CNC units, having a ∼0.31 nm cross-section by a size-sieving mechanism. PEG dispersion at 21 wt %: (i) enlarges to ∼0.52 nm the cross-sectional size of part of these nanocavities, increasing the permeability of N_2_, CO_2_, and CH_4_, and (ii) changes the molecular transport mechanism to configurational diffusion. Low molecular weight PEG400 shows a high dispersion degree between the CNC units and ensures the largest membrane permeability values, promoting the formation of an extended network of enlarged nanochannels. Nanochannel engineering can be done by changing the PEG molecular size and permits tuning the CO_2_ selectivity of these filler-dominated nanocomposite membranes. A fraction of high molecular weight PEG8000 and PEG20000 forms PEG nanoaggregates, ensuring improved CO_2_ selectivity due to the presence of CO_2_-selective sites on the PEG nanoaggregate surface but lowers the molecular transport rates.

## Supplementary Material



## Notes

Methods and procedures for gas transport tests. Methods and procedures for PALS analysis. Tables including PALS data reported in the text. SEM micrographs, DSC thermograms, and gas transport data of neat CNC membranes (PDF)
