# Supplementary material for: Filler-Dominated Polymer Nanocomposites: Molecular Transport in Extended Nanocavities
Source: J Phys Chem C Nanomater Interfaces. 2026 Jul 6;130(28):9898–907. doi: 10.1021/acs.jpcc.6c01646 (PMC13384021; doi:10.1021/acs.jpcc.6c01646)
Supplement: Supplementary file 1 [file jp6c01646_si_001.pdf]

Filler- dominated polymer nanocomposites: molecular transport in extended nanocavities

E. D'Amato,[&] M. Scarpa,[&] R. S. Brusa,[&,%] P. Battocchio,[&] A. Wagner,[(@)] M. O. Liedke,[(@)] E. Hirschmann,[(@)] P. Mengucci,[(£)] A. Korn[($)] and R. Checchetto[&,%]

[(&)] Department of Physics, University of Trento, Via Sommarive 14, 38123 Povo, Trento, Italy.

[(@)] Helmholz-Zentrum Dresden-Rossendorf, Institute of Radiation Physics, Dresden, Germany, Bautzner Landstr. 400, 01328 Dresden, Germany.

[(%)] TIPFA-INFN Trento, Vai Sommarive 14, 38123 Povo, Trento, Italy.

[(£)] Dept. SIMAU, Faculty of Engineering, Università Politecnica delle Marche (UNIVPM), Via Brecce Bianche 12, 60132 Ancona, Italy.

[(§)] Department of Physics, Chemnitz University of Technology, Reichenhainer Str. 70, 09126 Chemnitz, Germany.

Supplementary Information on PALS tests.

Variable energy positron annihilation lifetime spectroscopy (VEPALS) measurements were conducted on at the Mono-energetic Positron Source (MePS) beamline at HZDR, Germany [1]. A $CeBr_3$ scintillator detector coupled to a Hamamatsu R13089-100 photomultiplier tube (PMT) was utilized for gamma photons detection. The signals were processed by the Teledyne SP Devices ADQ14DC-2X digitizer (14 bit vertical resolution and 2GS/s horizontal resolution) [2]. The overall time resolution of the measurement system was 0.230-0.250 ns and all spectra contained at least $1\cdot10^7$ counts. A typical lifetime spectrum $N(t)$, the absolute value of the time derivative of the positron decay spectrum, is described by

$$N(t) = R(t) * \sum_{i=1}^{k+1} \frac{I_i}{\tau_i} e^{\frac{-t}{\tau_i}} + background$$

where k is the number of different defect types contributing to the positron trapping, which are related to $k + 1$ components in the spectra with the individual lifetimes τ_i and intensities I_i
$(\sum I_i) = 1$ [3].

The instrument resolution function $R(t)$ is a sum of two Gaussian functions with distinct intensities and relative shifts both depending on the positron implantation energy, E_p. It was determined by the measurement and analysis of a reference sample, i.e. Yttria-stabilized zirconia (YSZ), which exhibited a single well-known lifetime component.

All the spectra were deconvoluted using a non-linear least-squares fitting method, minimized by the Levenberg-Marquardt algorithm, employed within the fitting software package PALSfit [4] into 4 major lifetime components, which directly evidence para-Positronium and bulk annihilation localized annihilation (τ_1), annihilation at point defects (τ_2) and at 2 different void types (sizes; τ_3 and τ_4). Their relative intensities scale typically with concentration of each defect type. In general, positron lifetime increases with defects and free volume size. The positron lifetime and its intensity has been probed in function of positron implantation energy E_p which was recalculated to the mean implantation depth $\langle z_p \rangle$.

[1] A. Wagner, M. Butterling, M.O. Liedke, K. Potzger, R. Krause-Rehberg, *Positron annihilation lifetime and Doppler broadening spectroscopy at the ELBE facility*, in: AIP Conf. Proc., 2018: p. 040003. https://doi.org/10.1063/1.5040215.

[2] E. Hirschmann, M. Butterling, U. Hernandez Acosta, M.O. Liedke, A.G. Attallah, P. Petring, M. Görler, R. Krause-Rehberg, A. Wagner, *A new system for real-time data acquisition and pulse parameterization for digital positron annihilation lifetime spectrometers with high repetition rates*, J. Instrum. 16 (2021) P08001. https://doi.org/10.1088/1748-0221/16/08/P08001.

[3] R. Krause-Rehberg, H.S. Leipner, *Positron annihilation in semiconductors: defect*

studies, 1999.

[4] J. V. Olsen, P. Kirkegaard, N.J. Pedersen, M. Eldrup, PALSfit: *A new program for the evaluation of positron lifetime spectra*, Phys. Status Solidi. 4 (2007) 4004–4006. https://doi.org/10.1002/pssc.200675868.

Other references

E. des Ligneris, D. Samélor, A. Sekkat, M.O. Liedke, S. Klug, C. Josse, T. Hungria, A. Wagner and B. Caussat, *Nanoporous alumina-silica nanolaminates grown by mixed thin films processes for selective environmental barrier layers on carbon surfaces*, Appl. Surf. Sci. **719** (2026) 165144.

I. Baert, S. Eyley, R. Helm, M. Dickmann, M. Butterling, M.O. Liedke, A. Wagner, W. Thielemans, I.F.J. Vankelecom and R. Verbeke, *The impact of densification steps on epoxide-based TFC membranes*, Sep. Purif. Technol. **381** (2026) 135570.

M. Biesuz, A.M. Abebe, L. Karacasulu, S. Mariazzi, R.S. Brusa, D. Szewczyk, N. Pugno, G. Sero, M. Butterling, M.O. Liedke, A. Wagner, E. Hirschmann, M. Cassetta and G.D. Sorarù, *From polymer to SiOC glass: structure, microstructure, mechanical, and thermal properties*, J. Am. Ceram. Soc. **108** (2025) e20708.

A.G. Attallah, S. Prucnal, M. Buttering, E. Hirschmann, N. Koehler, S.E. Schulz, A. Wagner and M.O. Liedke, *Millisecond flash lamp curing for porosity generation in thin films*, Sci. Rep. **13** (2023) 7765.

J. Franke, M.O. Liedke, A. Schäfer, M. Butterling, A.G. Attallah, E. Hirschmann, A. Wagner and R. Dahlmann, *Comparison of the Coating Structure of Silicon-Based PECVD Coatings With Varying Organic Content*, Plasma Process. Polym. (2025) e70107.

Positron Annihilation Lifetime Spectroscopy data

Additional PALS data are available upon request to the correspondig author.

$E\ (keV)$<br>neat CNC	$\tau_1\ (ns)$<br>$I_1\ (\%)$	$\tau_2\ (ns)$<br>$I_2\ (\%)$	$\tau_3\ (ns)$<br>$I_3\ (\%)$
1	0.25 (F)<br>26.5 $\pm$ 0.5	0.413 $\pm$ 0.002<br>59.8 $\pm$ 0.4	1.36 $\pm$ 0.01<br>13.7 $\pm$ 0.1
3	0.25 (F)<br>26.0 $\pm$ 0.5	0.406 $\pm$ 0.002<br>59.3 $\pm$ 0.4	1.25 $\pm$ 0.01<br>14.7 $\pm$ 0.2
6	0.25 (F)<br>25.7 $\pm$ 0.5	0.408 $\pm$ 0.002<br>60.0 $\pm$ 0.4	1.26 $\pm$ 0.01<br>14.1 $\pm$ 0.1
11	0.25 $\pm$ 0.01<br>25.0 $\pm$ 0.5	0.404 $\pm$ 0.002<br>61.0 $\pm$ 0.5	1.254 $\pm$ 0.001<br>14.0 $\pm$ 0.1

Table S1: neat CNC films, data obtained at 23°C. (F): fixed value

$E\ (keV)$<br>CNC/PEG400	$\tau_1\ (ns)$<br>$I_1\ (\%)$	$\tau_2\ (ns)$<br>$I_2\ (\%)$	$\tau_3\ (ns)$<br>$I_3\ (\%)$	$\tau_4\ (ns)$<br>$I_4\ (\%)$
1	0.2 (F)<br>15.0 $\pm$ 0.5	0.375 $\pm$ 0.003<br>67.8 $\pm$ 0.3	1.21 $\pm$ 0.05<br>11.8 $\pm$ 0.3	3.0 $\pm$ 0.1<br>4.3 $\pm$ 0.5
3	0.2 (F)<br>14.6 $\pm$ 0.4	0.375 $\pm$ 0.002<br>66.9 $\pm$ 0.3	1.23 $\pm$ 0.03<br>12.7 $\pm$ 0.2	3.10 $\pm$ 0.07<br>5.6 $\pm$ 0.3
6	0.2 (F)<br>15.6 $\pm$ 0.4	0.378 $\pm$ 0.002<br>65.4 $\pm$ 0.3	1.22 $\pm$ 0.03<br>11.9 $\pm$ 0.3	3.01 $\pm$ 0.03<br>7.1 $\pm$ 0.2
11	0.2 (F)<br>14.3 $\pm$ 0.2	0.372 $\pm$ 0.002<br>66.0 $\pm$ 0.3	1.18 $\pm$ 0.03<br>12.9 $\pm$ 0.2	2.95 $\pm$ 0.03<br>7.7 $\pm$ 0.2

Table S2: CNC/PEG400 films, data obtained at 23°C. (F): fixed value.

$E\ (keV)$<br>CNC/PEG8000	$\tau_1\ (ns)$<br>$I_1\ (\%)$	$\tau_2\ (ns)$<br>$I_2\ (\%)$	$\tau_3\ (ns)$<br>$I_3\ (\%)$	$\tau_4\ (ns)$<br>$I_4\ (\%)$
1	0.2 (F)<br>12.5 $\pm$ 0.5	0.375 $\pm$ 0.002<br>70.9 $\pm$ 0.3	1.18 $\pm$ 0.04<br>11.4 $\pm$ 0.3	3.0 $\pm$ 0.1<br>5.1 $\pm$ 0.4
3	0.2 (F)<br>13.7 $\pm$ 0.4	0.379 $\pm$ 0.002<br>68.4 $\pm$ 0.3	1.20 $\pm$ 0.03<br>11.7 $\pm$ 0.2	2.91 $\pm$ 0.04<br>6.2 $\pm$ 0.3
6	0.2 (F)<br>12.6 $\pm$ 0.4	0.375 $\pm$ 0.002<br>69.1 $\pm$ 0.3	1.14 $\pm$ 0.03<br>11.2 $\pm$ 0.2	2.76 $\pm$ 0.04<br>7.1 $\pm$ 0.3
11	0.2 (F)<br>13.2 $\pm$ 0.4	0.376 $\pm$ 0.002<br>68.8 $\pm$ 0.2	1.17 $\pm$ 0.03<br>11.4 $\pm$ 0.2	2.85 $\pm$ 0.05<br>6.6 $\pm$ 0.3

Table S3: CNC/PEG8000 films, data obtained at 23°C. (F): fixed value.

$E\ (keV)$<br>CNC/PEG20000	$\tau_1\ (ns)$<br>$I_1\ (\%)$	$\tau_2\ (ns)$<br>$I_2\ (\%)$	$\tau_3\ (ns)$<br>$I_3\ (\%)$	$\tau_4\ (ns)$<br>$I_4\ (\%)$
1	0.2 (F)<br>12.7 ± 0.4	0.376 ± 0.002<br>71.7 ± 0.3	1.22 ± 0.03<br>11.5 ± 0.1	3.16 ± 0.07<br>4.1 ± 0.2
3	0.2 (F)<br>11.5 ± 0.5	0.372 ± 0.002<br>70.5 ± 0.3	1.18 ± 0.04<br>11.3 ± 0.2	2.85 ± 0.04<br>6.7 ± 0.3
6	0.2 (F)<br>12.9 ± 0.5	0.378 ± 0.002<br>69.6 ± 0.3	1.15 ± 0.04<br>10.6 ± 0.2	2.73 ± 0.05<br>7.0 ± 0.3
11	0.2 (F)<br>12.6 ± 0.4	0.377 ± 0.002<br>69.9 ± 0.3	1.16 ± 0.04<br>10.7 ± 0.2	2.76 ± 0.05<br>6.8 ± 0.3

Table S4: CNC/PEG20000 films, data obtained at 23°C. (F): fixed value.

CNC/PEG400<br>$11\ keV$	$\tau_1\ (ns)$<br>$I_1\ (\%)$	$\tau_2\ (ns)$<br>$I_2\ (\%)$	$\tau_3\ (ns)$<br>$I_3\ (\%)$	$\tau_4\ (ns)$<br>$I_4\ (\%)$
23°C	0.2 (F)<br>14.3 ± 0.2	0.372 ± 0.002<br>66.0 ± 0.3	1.18 ± 0.03<br>12.9 ± 0.2	2.95 ± 0.03<br>47.7 ± 0.2
40°C	0.2 (F)<br>13.6 ± 0.4	0.374 ± 0.002<br>67.1 ± 0.3	1.23 ± 0.01<br>12.0 ± 0.2	2.96 ± 0.03<br>7.2 ± 0.2
60°C	0.2 (F)<br>12.5 ± 0.5	0.368 ± 0.002<br>67.3 ± 0.3	1.13 ± 0.04<br>11.8 ± 0.2	2.83 ± 0.04<br>8.3 ± 0.3
80°C	0.2 (F)<br>13.3 ± 0.4	0.373 ± 0.002<br>67.3 ± 0.3	1.20 ± 0.04<br>11.3 ± 0.2	2.90 ± 0.05<br>8.1 ± 0.2

Table S5: CNC/PEG400 films. (F): fixed value.

Supplementary Information on Permeations Tests

Gas transport tests were carried out by gas phase permeation in single gas conditions and dead-end configuration using disc- shaped samples with 13 mm diameter. We used the following procedure. Before each transport test, the feed chamber is evacuated to final pressure ~ 10^{-3} mbar. The permeate chamber is evacuated by a high-pumping speed turbomolecular pump to a final pressure in the low 10^{-8} mbar range. At time $t = 0$, the feed side of the membrane was exposed to the test gas α at pressure p_{HPS} ~ 10^{3} mbar while the other side faced the continuously pumped permeate chamber. The permeation flux $f_{\alpha,exp}(t)$ of the α gas specie, was monitored by measuring its partial pressure

$p_\alpha(t)$ in the permeate chamber by a calibrated Quadrupole Mass Spectrometer and calculated according to the relationship:

$$f_{\alpha,exp}(t) = \frac{1}{A}\frac{1}{RT_{ch}} s_\alpha p_\alpha(t) \qquad (1)$$

where A is the membrane surface area, R the universal gas constant, T_{ch} the temperature of the vacuum chamber and s_α the pumping speed for the α gas specie.

Under the conditions of the solution-diffusion model, the permeation process of the test gas α is controlled by two parameters, gas diffusivity D_α (cm^2 / s) and gas solubility S_α [cm^3(STP) / cm^3 cmHg], and the theoretical permeation flux $f_\alpha(t)$ is given by:

$$f_\alpha(t) = \frac{D_\alpha S_\alpha}{L} p_{HPS} \left[1 + 2\sum_{n\geq 1} (-1)^n \, exp(-\frac{D_\alpha n^2 \pi^2 t}{L^2})\right] \qquad (2)$$

where the parameter $P_\alpha = D_\alpha S_\alpha$ is the permeability of the membrane to the α test gas. The gas transport parameters P_α and D_α were obtained fitting the experimental $f_{\alpha,exp}(t)$ curve in transient and stationary transport conditions with eqn. (2), see Figure S1. The effective gas solubility S_α is then obtained by the relation $S_\alpha = \frac{P_\alpha}{D_\alpha}$. The main contribution to their experimental uncertainty is given by the uncertainty δL of the membrane sample thickness L.

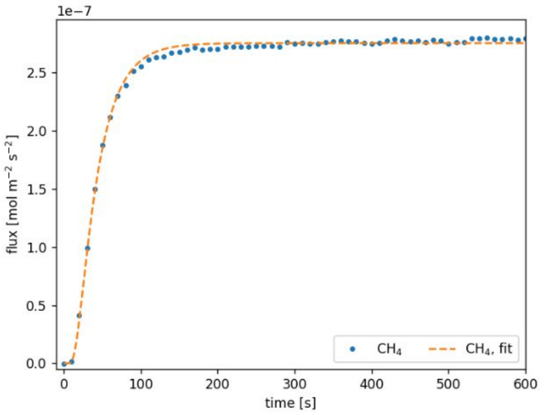

Figure S1. Points: CH_4 permeation curve $f_{\alpha,exp}(t)$ (sampled data) obtained in permeation tests carried out with the CNC/PEG400 nanocomposite membrane at $T = 30 \pm 1$ °C and feed pressure $p_{HPS} = 1.00 \pm 0.05$ bar. Dashed line: $f_{\alpha}(t)$ fitting curve by eq. 2.

R. Checchetto, *Accurate monitoring of gas mixture transport kinetics through polymeric membranes*, Separ. Purif. Technol. **277** (2021) 119477.

R. Checchetto, M. Scarpa, M.G. De Angelis and M. Minelli, *Mixed gas diffusion and permeation of ternary and quaternary $CO_2/CO/N_2/O_2$ gas mixtures in Matrimid®, polyetherimide and poly(lactic acid) membranes for CO_2/CO separation*, Journal of Membrane Science **659** (2022) 120768.

R. Checchetto, T. Facchinelli, G. Cantalini and M. Scarpa, *Tunable gas selectivity of cellulose nanocrystals – Polyethylene glycol composite membranes*, Int. J. Hydrogen Energy **57** (2024) 688–695.

R. Checchetto, *Experimental study on the transport of light gas molecules through low-density polyethylene films*, Int. J. Polym. Sci. (2018) Article ID 4903904.

D. Roilo, C. A. Maestri, M. Scarpa, P. Bettotti, W. Egger, T. Koschine, R. S. Brusa and R. Checchetto, *Cellulose Nanofibrils Films: Molecular Diffusion through Elongated Sub-Nano Cavities*, J. Chem. Phys. C **121** (2017) 15437−15447.

D. Roilo, C. A. Maestri, M. Scarpa, P. Bettotti and R. Checchetto, *Gas barrier and optical properties of cellulose nanofibers coatings with dispersed TiO_2 nanoparticles,* Surf. Coat. Technol. **343** (2018) 131-137.

Supplementary information of neat CNC membrane samples.

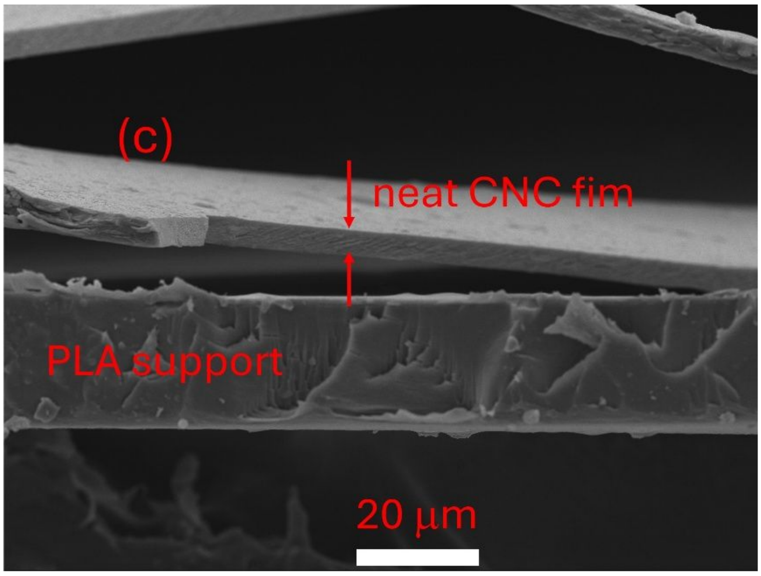

Figure S2: SEM micrograph of the cross-section of the CNC film grown on PLA support. To analyze the CNC/PLA cross-section, samples were previously freeze-cut in liquid nitrogen.

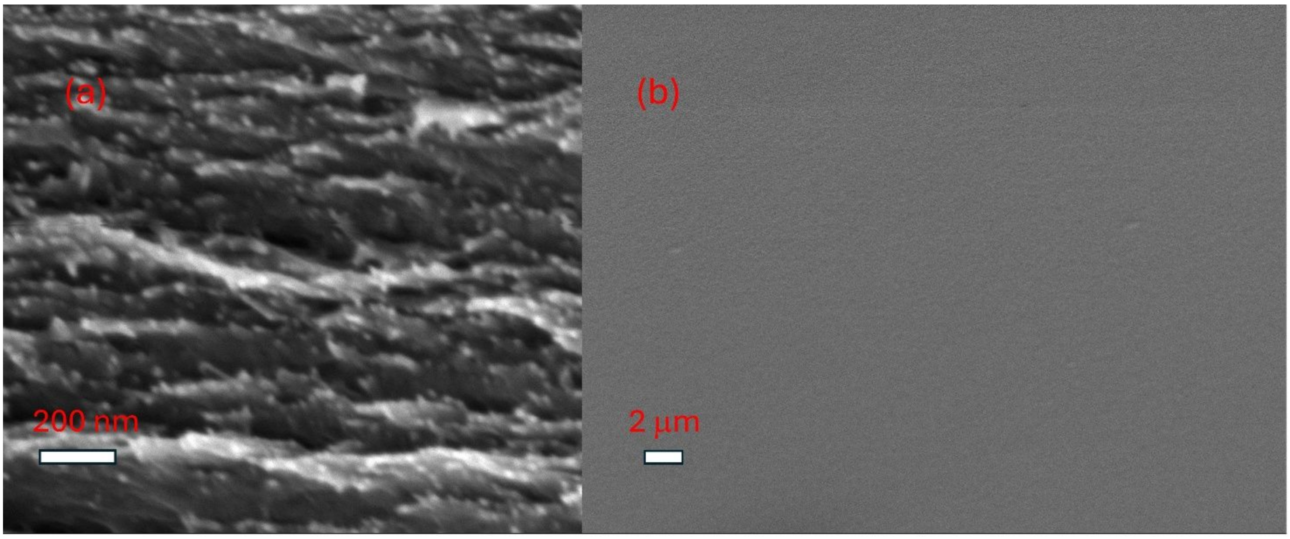

Figure S3: (a) SEM micrograph of the cross-section of the neat CNC film. (b) SEM micrograph of the surface of the neat CNC film.

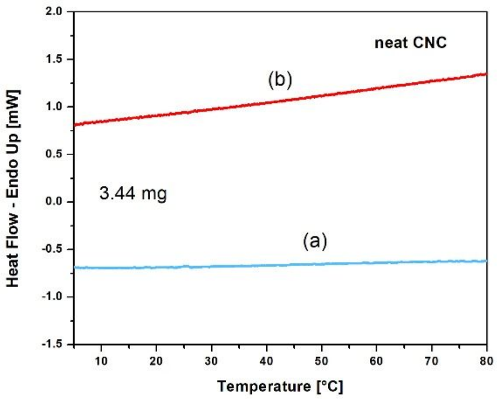

Figure S4: DSC thermograms of the neat CNC membrane. Line (a): first cooling run. Line (b) the second heating run. The sample mass is also reported.

Gas transport data of neat CNC membrane:

Deuterium:

$$\Phi = (0.10 \pm 0.03) \frac{mL\ \mu m}{m^2\ day\ kPa}$$

$$D = (2.2 \pm 0.4) \times 10^{-10} \frac{cm^2}{s},$$

Helium:

$$\Phi = (0.4 \pm 0.1) \frac{mL\ \mu m}{m^2\ day\ kPa}$$

$$D = (4.4 \pm 0.8) \times 10^{-9} \frac{cm^2}{s}$$

No permeation signal was observed through the neat CNC membranes with the following test gases: O_2, N_2, CO_2, CH_4
